# Supplementary material for: Deletion of wfs1 Impairs Oligodendrocyte Precursor Cells Dorsal Distribution and Myelination Through the wfs1-hmgcs1 Axis in Zebrafish
Source: Biology (Basel). 2026 Aug 16;15(16):1402. doi: 10.3390/biology15161402 (PMC13510151; doi:10.3390/biology15161402)
Supplement: Supplementary file 1 [file biology-15-01402-s001.zip › File S1-Transcriptome sequencing data and differentially expressed genes (DEGs) in wfs1-- zebrafish at 5 dpf/2.KEGG/wfs1KOvsWT/ALL/src/dre00640.html]

dre00640


- dre:559403

- Up regulated genes

559403\_1(1.4336766768898)

- dre:437019

- Up regulated genes

437019\_1(2.62419743690105)

- dre:317746

- Up regulated genes

317746(2.4778823913538)

- dre:317746

- Up regulated genes

317746(2.4778823913538)

Close
